# Analysis of Brain Functional Connectivity Neural Circuits in Children With Autism Based on Persistent Homology

**DOI:** 10.3389/fnhum.2021.745671

**Published:** 2021-09-13

**Authors:** Di Liang, Shengxiang Xia, Xianfu Zhang, Weiwei Zhang

**Affiliations:** ^1^School of Science, Shandong Jianzhu University, Jinan, China; ^2^School of Control Science and Engineering, Shandong University, Jinan, China

**Keywords:** ASD, resting-state fMRI, FC neural circuits, persistent homology, VR complex

## Abstract

Autism spectrum disorder (ASD) is a complex neuropsychiatric disorder with a complex and unknown etiology. Statistics demonstrate that the number of people diagnosed with ASD is increasing in countries around the world. Currently, although many neuroimaging studies indicate that ASD is characterized by abnormal functional connectivity (FC) patterns within brain networks rather than local functional or structural abnormalities, the FC characteristics of ASD are still poorly understood. In this study, a Vietoris-Rips (VR) complex filtration model of the brain functional network was established by using resting-state functional magnetic resonance imaging (fMRI) data of children aged 6–13 years old [including 54 ASD patients and 52 typical development (TD) controls] from the Autism Brain Imaging Data Exchange (ABIDE) public database. VR complex filtration barcodes are calculated by using persistent homology to describe the changes in the FC neural circuits of brain networks. The number of FC neural circuits with different length ranges at different threshold values is calculated by using the barcodes, the different brain regions participating in FC neural circuits are discussed, and the connectivity characteristics of brain FC neural circuits in the two groups are compared and analyzed. Our results show that the number of FC neural circuits with lengths of 8–12 is significantly decreased in the ASD group compared with the TD control group at threshold values of 0.7, 0.8 and 0.9, and there is no significant difference in the number of FC neural circuits with lengths of 4–7 and 13–16 and lengths 16. When the thresholds are 0.7, 0.8, and 0.9, the number of FC neural circuits in some brain regions, such as the right orbital part of the superior frontal gyrus, the left supplementary motor area, the left hippocampus, and the right caudate nucleus, involved in the study is significantly decreased in the ASD group compared with the TD control group. The results of this study indicate that there are significant differences in the FC neural circuits of brain networks in the ASD group compared with the TD control group.

## 1. Introduction

Autism spectrum disorder (ASD), also known as autism, is a complex neurodevelopmental disorder. It usually develops before the age of 3 years and includes the typical clinical features of impaired language communication and social communication skills, a narrow range of interests, and repetitive and stereotyped behaviors (American-Psychiatric-Association, 2013). In 2019, the “China autism education rehabilitation III industry development status report” showed that there are more than 10 million autistic people in China, including more than 2 million children with ASD, and the number is increasing at a rate of ~2% annually. According to the Centers for Disease Control and Prevention (CDC), one out of every 59 children in the U.S. was diagnosed with ASD in 2018. Statistics demonstrate that the number of people diagnosed with ASD is increasing worldwide. The etiology of ASD is complex, the pathogenesis is still unclear, and the early symptoms are not typical. Over the years, the clinical diagnosis of ASD has been mainly based on typical clinical symptoms, the Diagnostic and Statistical Manual of Mental Disorders (Fifth Edition) and the Childhood Autism Rating Scale. Identification and diagnosis are subjective and less stable. Professor Laurent Mottron's research team (Rdgaard et al., 2019) found that the objective performance differences between individuals with and without autism are actually narrowing due to changes in and the updating of diagnostic practices. Therefore, it is becoming increasingly important to carry out quantitative assisted diagnosis through the study of the neurophysiological basis of ASD.

The emergence of functional magnetic resonance imaging (fMRI) has brought new developments to cognitive neuroscience. Functional connectivity (FC) is described as the temporal correlation of a neurophysiological index measured in different brain regions (Friston et al., 1993). FMRI realizes functional brain imaging by examining magnetic field changes caused by the blood oxygen level-dependent (BOLD) response entering brain cells (Ogawa et al., 1990). In the resting state, spontaneous neural activities in spatially separated brain regions involved in the execution of separate cognitive functions, such as default mode and executive control, are highly correlated (Salvador et al., 2005; He et al., 2009). Resting-state FC can refer to the relationship between neuronal activation patterns in brain regions and reflect the information exchange between brain regions (Cole et al., 2016; Tomasi and Volkow, 2019). Research (Lee et al., 2013; Xu et al., 2013) on resting-state fMRI can reveal the functional integration characteristics, dynamic change rules of spontaneous brain activities, and topological properties of the FC neural circuits of brain networks.

A considerable amount of neuroimaging literature (Cherkassky et al., 2006; Kennedy and Adolphs, 2012; Martino et al., 2014) indicates that ASD is characterized by abnormal overall connectivity patterns within brain networks. Just et al. (2004) believed that the deficit in verbal information-integration ability in ASD patients is related to decreased functional brain connectivity. Wicker et al. (2008) believes that abnormalities in FC among the amygdala, prefrontal cortex, and posterior association cortex are the main obstacles for integrating emotional information in ASD patients. Zhang (2015) believed that the deficits in controlling and regulating attention and alertness of ASD patients may be caused by damage to the bottom-up neural circuits with the basal nucleus of the amygdala-primary visual cortex. Analysis of brain FC networks (Kana et al., 2011; Supekar et al., 2013) based on resting-state fMRI will provide an important biological basis for the diagnosis and treatment of ASD from the perspective of FC.

Persistent homology, a newly developed method in topological data analysis (Carlsson, 2009), is an effective tool for analyzing high-dimensional nonlinear data and exploring its nonlinear structures. In fact, the concept of persistence was introduced by Robins (1999), and refined by Edelsbrunner et al. (2002) and Zomorodian and Carlsson (2005). The most important advantage of persistent homology is that it is not restricted by threshold values. Persistent homology has been applied in many kinds of data (including brain data) as described in the section 1 (Carlsson, 2020). For example, the research results of Victor et al. (2018) show that the persistent homology method is a powerful new tool for analyzing brain networks; Lee et al. (2018) used persistent homology to detect abnormal holes in brain network connectivity and provided another representation of the shape of brain networks; Pereira and de Mello (2015) developed a framework for clustering time-series and spatial data based on topological properties, which could detect similarities in recurrent behavior for time series data sets and spatial structures in spatial data sets, such as electroencephalography data from alcoholic subjects; Kanari et al. (2018) introduced the Topological Morphology Descriptor, derived from principles of persistent homology, which could reliably describe the shape of neurons and advance our understanding of the anatomy and diversity of the neuronal morphologies; Cassidy et al. (2015) have developed a new distance measure to discriminate resting state fMRI connectivity using persistent homology. In this study, with the resting-state fMRI data of some children aged 6–13 from the “Autism Brain Imaging Data Exchange (ABIDE)” public database, Vietoris-Rips (VR) complex filtration models were established for ASD patients and typical development (TD) controls based on their brain FC. The barcodes of VR complex filtration were calculated by the persistent homology method. Therefore, FC neural circuits of ASD patients and TD controls at different threshold values can be obtained from the barcodes. The change rules of FC neural circuits in ASD patients are found by analyzing the number of FC neural circuits of different length ranges and brain regions involved at different threshold values; furthermore, it is expected to provide objective biological markers for the early diagnosis of ASD.

## 2. Materials and Methods

### 2.1. Data Acquisition

The resting-state fMRI data used in this paper are from the four collection sites, ABIDEII-NYU_1, ABIDEII-NYU_2, ABIDEII-SU_2, and ABIDEII-EMC_1, in the ASD Public Dataset (ABIDE) (Martino and Milham, 2017). The imaging data conditions of the ASD group and the TD control group are as follows: (1) there (Moran et al., 2014) was a strong gender effect in ASD patients, so the subjects were all male; (2) all subjects were dextral; (3) there was an age effect on brain FC, so the age of the subjects was 6–13 years old, including 54 ASD patients [mean (9.3 ± 1.9) years old] and 52 TD controls [mean (9.6 ± 1.9) years old], and there was no significant difference in age between the two groups (*p* = 0.31).

### 2.2. Resting-State fMRI Processing

In this study, the tool DPABI (Yan et al., 2016) (http://rfmri.org/DPABI) is used for preprocessing resting-state fMRI data. The preprocessing of data mainly includes the following steps: (1) Removing the first 10 items from the time series and realigning; (2) Cleaning low frequency scanner drift and applying bandpass filtering (0.01–0.1 Hz); (3) Normalizing spatial data by DARTEL; (4) Selecting anatomical automatic labeling (AAL) (Tzourio-Mazoyer et al., 2002) to define regions of interest (ROIs); and (5) Calculating the FC matrix based on BOLD time series for each pair of brain regions. For more data preprocessing details, please refer to the Supplementary Material.

### 2.3. A VR Complex Filtration Model of Brain Networks

Let (*X, D*) be a metric space and a Vietoris-Rips (VR) complex with ε parameters be a simple complex denoted by VR(*X*, ε), whose vertex is the set *X*, and {*x*_0_, *x*_1_, ..., *x*_*k*_} generates a k-complex if and only if *d*(*x*_*i*_, *x*_*j*_) ≤ ε for all 0 ≤ *i*, *j* ≤ *k*.

A correlation matrix of the brain functional network is obtained by using DPABI. Each brain region is a vertex in the brain functional network. We use the Pearson correlation coefficient to define the distance between two vertices *P*_*i*_ and *P*_*j*_,$\;d_{X}\left(P_{i},P_{j}\right)=\sqrt{1-corr\left(P_{i},P_{j}\right)}$; therefore, the metric space (*X, d*_*X*_) is formed from the brain functional network. The correlation matrix is sorted from smallest to largest by the distance between the two vertices: ε_1_ < ε_2_ < … < ε_*M*_. We can obtain a VR filtration model of the brain network:


$$VR\left(X,\varepsilon_{1}\right)\subseteq VR\left(X,\varepsilon_{2}\right)\subseteq \cdots \subseteq VR\left(X,\varepsilon_{M}\right).$$

JavaPlex software is used to calculate the VR filtration barcode (the barcode-interval set). These intervals represent the lifetime of nontrivial cycles in the growing process of complexes. The left end of the interval represents the birth of a new topological property, and the right end of the interval represents its death. From this, we can describe the change rules of the Betti number of a brain network. The 0-dimensional Betti number β_0_ represents the number of components of the brain functional network, and the one-dimensional Betti number β_1_ represents the number of one-dimensional holes in the brain functional network, that is, the number of FC neural circuits in the brain network. VR complexes constructed from a point cloud data are described in the following example to help understand persistence homology.

**Figure 1:**
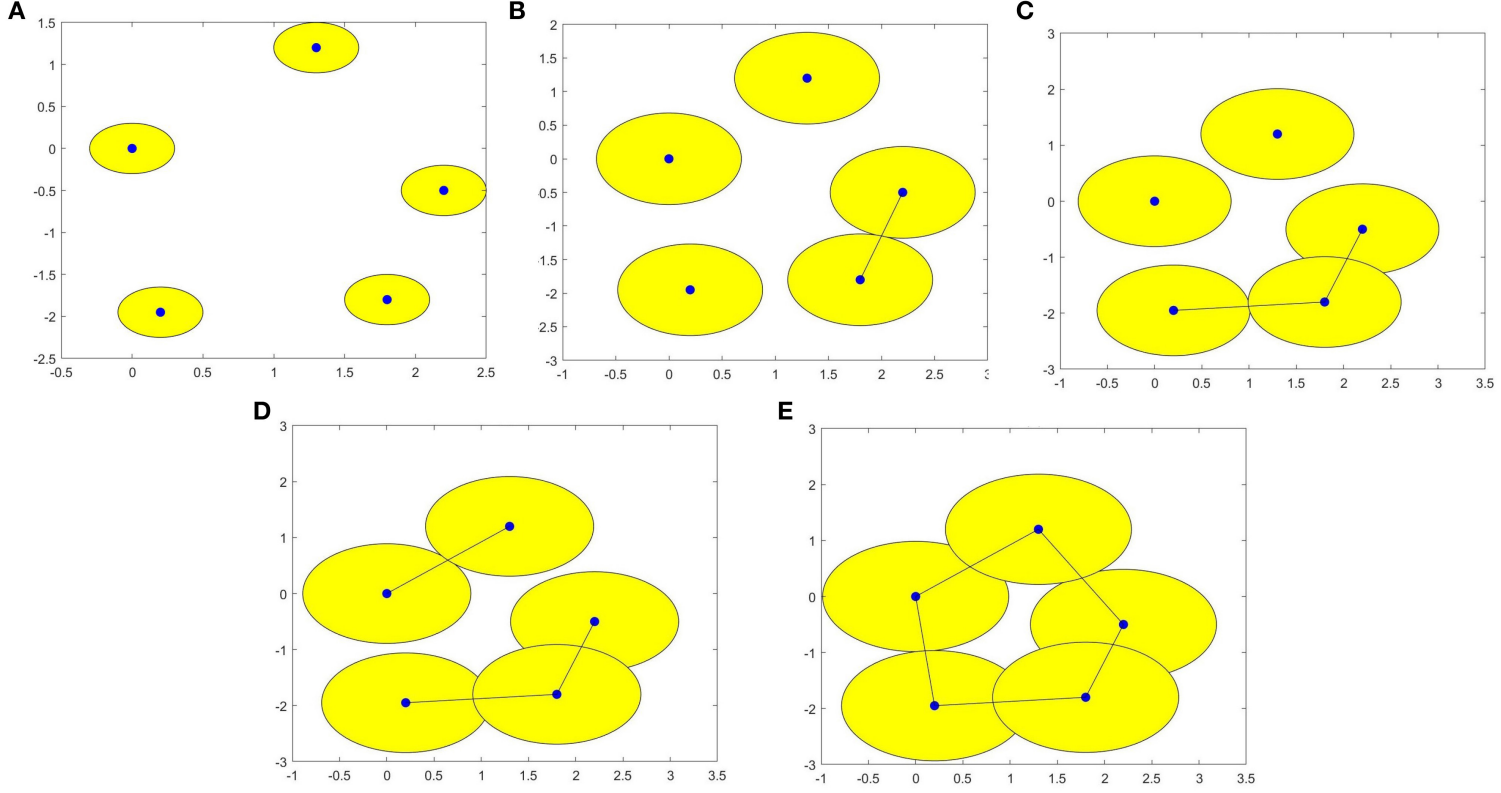
**(A–E)** are VR (X, ε) complexes of the point cloud data with ε = 0.60, 1.37, 1.62, 1.78, and 1.97 respectively.

**Figure 2:**
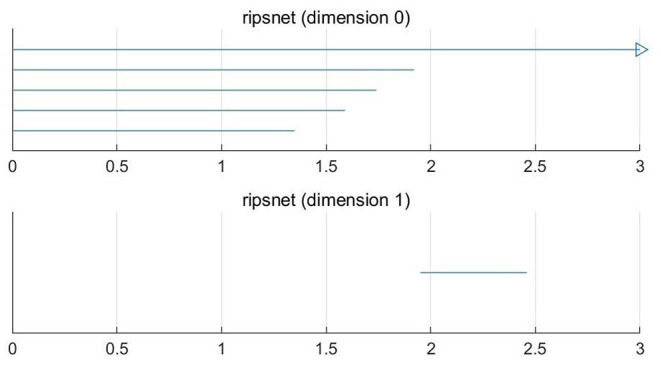
0-D and 1-D persistence barcodes of the point cloud data.

For a set *X* consisting of 5 points on the plane, we construct VR complexes at different values of ε. Let's draw five circles centered at the points with the radius ε/2. The VR complexes are shown in Figure 1. We explain each subfigure in detail to help understand Figure 1. In Figure 1A, there is no one-dimensional simplex formed at ε = 0.60. There are 5 components, this means β_0_ = 5. In Figure 1B, when the distance between the two points is less than ε = 1.37, the two lower right points form 1-dimensional simplex. There are 4 components, this means β_0_ = 4. In Figure 1C, the two bottom points are connected, and three bottom points form a new component when ε = 1.62, that is β_0_ = 3. In Figure 1D, in this case ε = 1.78, β_0_ = 2. In Figure 1E, a loop is formed, i.e., β_1_ = 1 and β_0_ = 1 when ε = 1.97. Then we calculate the persistence barcode according to persistence homology. The persistence barcodes of the data are shown in Figure 2.

**Figure 3:**
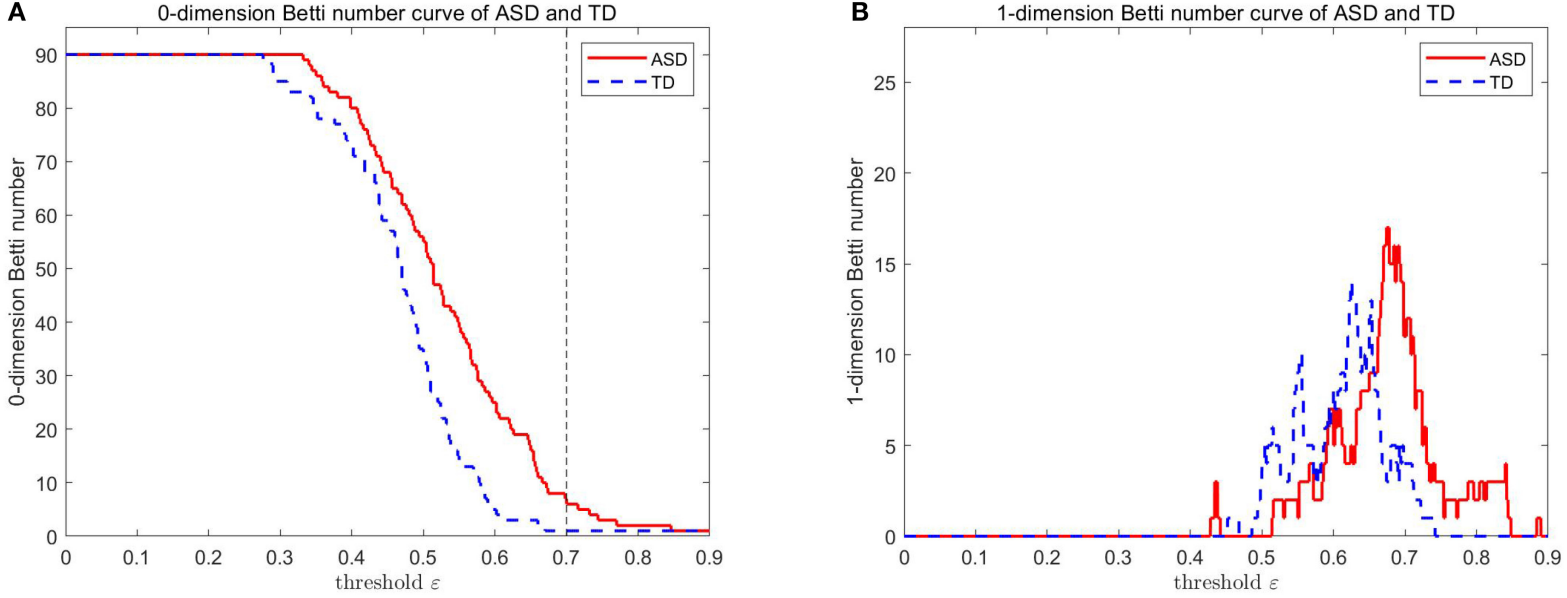
0-D and 1-D Betti number curves of the ASD brain network and the TD brain network.

We select AAL to define the ROIs, and a brain network has 90 vertices. Figure 3 presents the results obtained by calculating two original data points of an ASD patient and a TD control in ABIDE II with the above steps. A vertical line is drawn across the *x* axis, and the vertical coordinate of the intersection point of the vertical line and the 0-dimensional (1-dimensional) Betti curve is the 0-dimensional (1-dimensional) Betti number of the network corresponding to the threshold value. Figure 3 shows the curves of the 0-dimensional and 1-dimensional Betti numbers of VR filtration for the ASD patient and the TD control. It depicts the differences in the topological features of the ASD brain network and the TD brain network at different threshold values.

#### 2.3.1. Comparison of FC in Brain Networks

**Figure 4:**
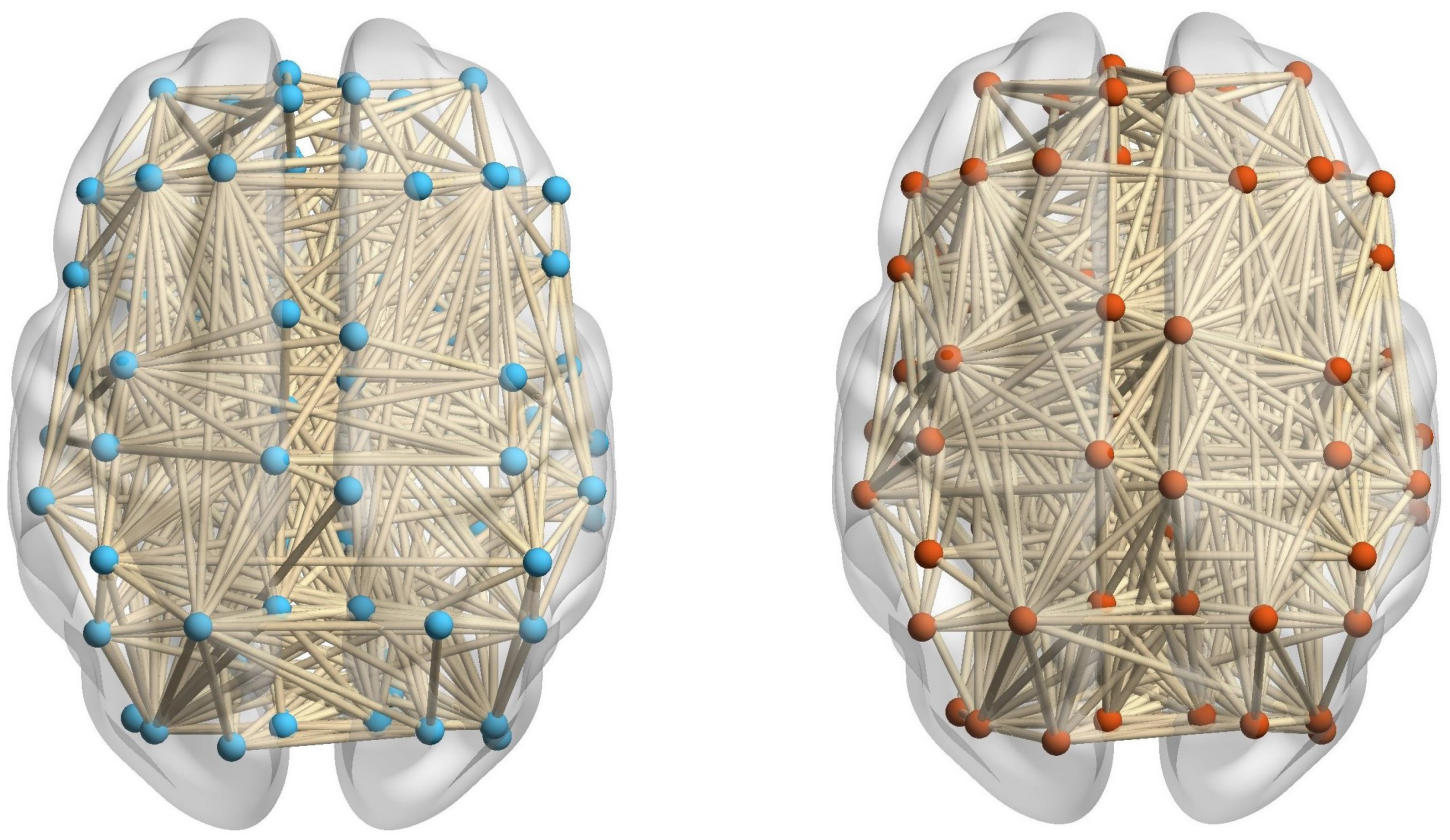
The ASD brain network **(left)** including 83 edges and the TD brain network **(right)** including 89 edges at a threshold value of 0.7.

The number of components of the brain network (i.e., β_0_) can be easily calculated from the 0-dimensional Betti curve. For example, the brain network based on AAL consists 90 brain regions. When the threshold value is 0.7, some brain regions are connected and form 83 edges, there are 7 components in the ASD patient. All brain regions are connected and form 89 edges, there is 1 component in the TD control. Figure 4 shows that there are more components in the ASD brain network than in the TD brain network. This can be interpreted as FC between brain regions of the ASD patient not being as tight as that of the TD control, and the deficit in FC between brain regions means that they cannot transmit information.

#### 2.3.2. Comparison of FC Neural Circuits in Brain Networks

**Figure 5:**
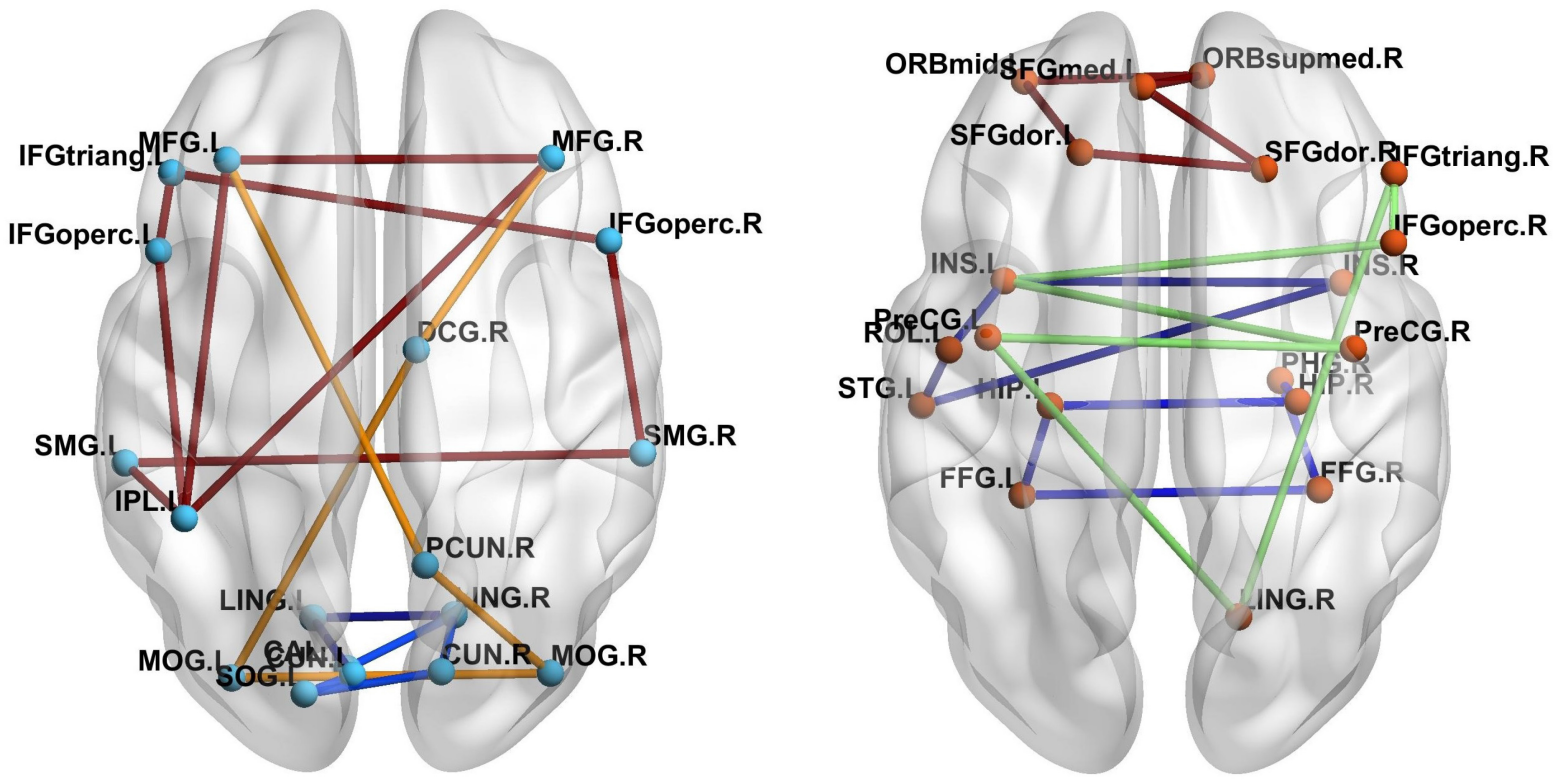
Comparison of the FC neural circuits in the ASD brain network **(left)** and the TD brain network **(right)** at a threshold value of 0.7. The abbreviations for names of brain regions in the AAL atlas are listed in the Supplementary Material. The differences of the colors of edges are just for easy to observe.

**Figure 6:**
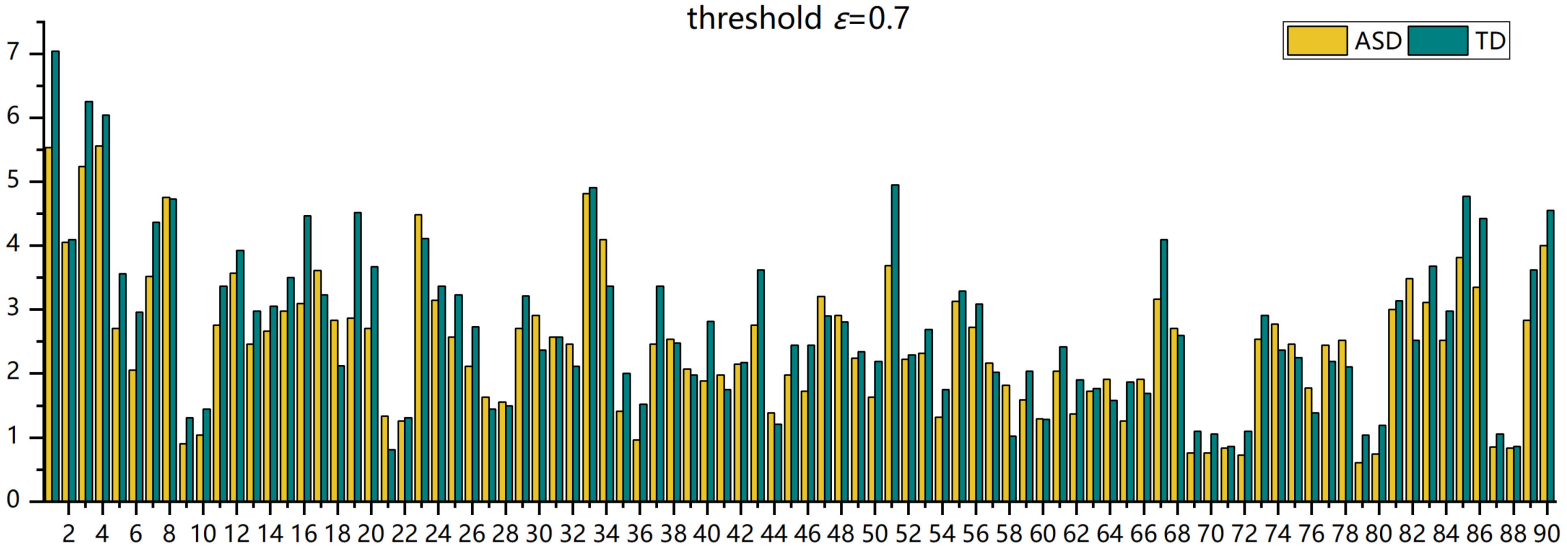
The histogram of the average number of FC neural circuits in each brain region in AAL in the ASD group and the TD control group.

The number of FC neural circuits in the brain network (i.e., β_1_) can be easily calculated from the one-dimensional Betti curve. For example, when the threshold value is 0.7, there are 32 FC neural circuits in the ASD patient and 51 FC neural circuits in the TD control. The number of FC neural circuits in the brain network at a threshold value of 0.7 means the total number of FC neural circuits formed in the brain network when the threshold value is no more than 0.7. Figure 5 shows part of the FC neural circuits in the ASD brain network and in the TD brain network. Figure 6 shows the average numbers of FC neural circuits in each brain region involved in the ASD group and the TD control group when the threshold value is 0.7. The brain regions and the number of brain regions involved in FC neural circuits are different. Differences in FC neural circuits may account for some of the dysfunction experienced by ASD patients.

## 3. Results

**Figure 7:**
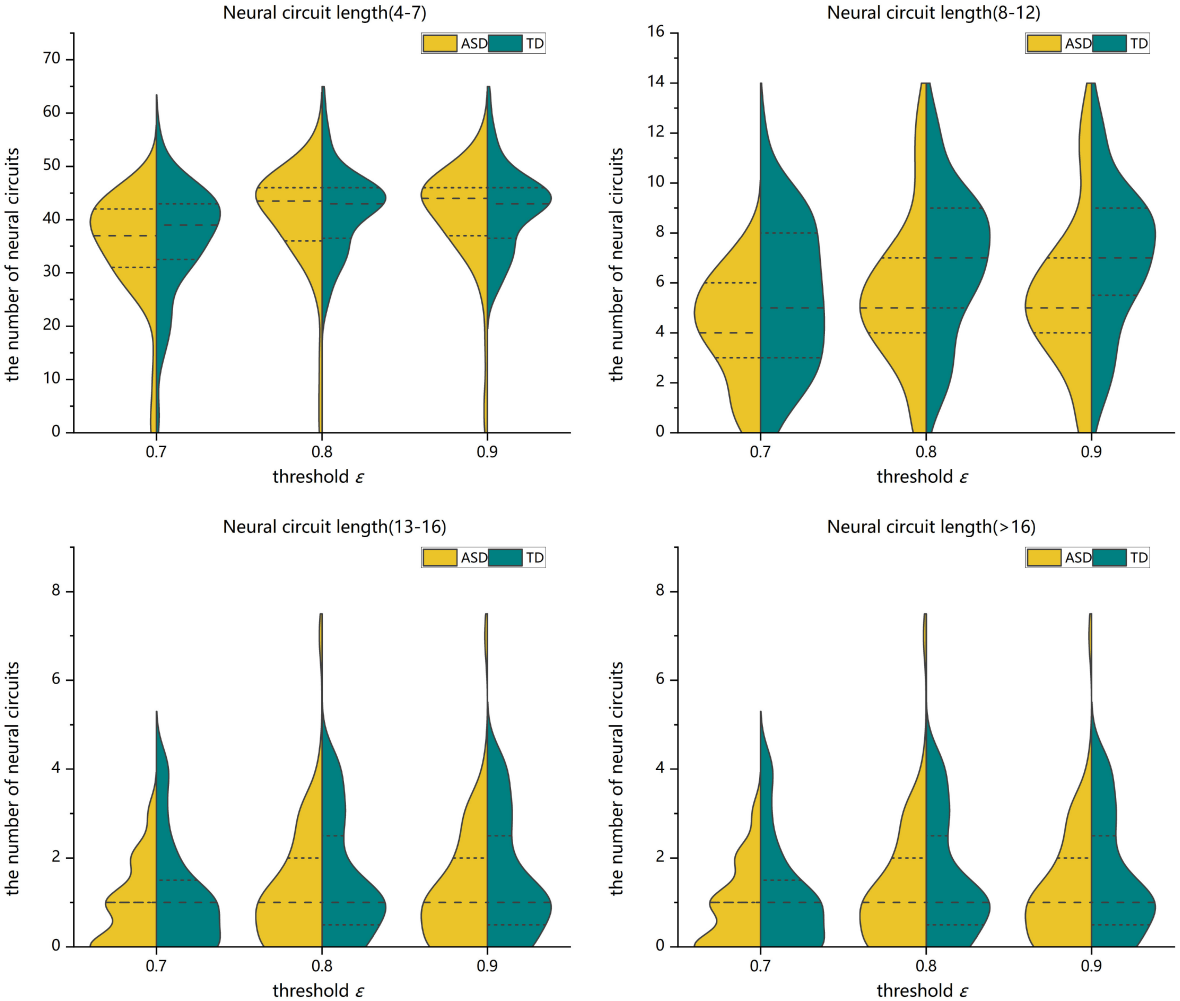
Violin pictures of the comparison of the numbers of FC neural circuits with different circuit length ranges of the two groups.

Two-sample Mann-Whitney *U*-tests are performed on the data from the ASD group and the TD control group to assess the group differences in the number of FC neural circuits with different circuit length ranges. FDR adjustments are performed. The statistical results show that when the threshold value is 0.7, 0.8, or 0.9, the number of FC neural circuits with a length of 8–12 is significantly decreased in the ASD group compared with in the TD control group, and there is no significant difference in the number of short neural circuits with a length of 4–7, long neural circuits with a length of 13–16, and ultralong neural circuits with a length 16 (see Figure 7 and Table 1).

**Table 1:** Statistically significant differences in the number of FC neural circuits (8–12) between the two groups.

**Threshold**	**ASD**	**TD**	***P*-value**	**FDR**
**value**	**patients **(*n* = 54)****	**controls **(*n* = 52)****		**adjustment**
0.7	4.19 ± 2.11	5.44 ± 2.87	0.027^*^↓	0.027^*^
0.8	5.48 ± 3.00	6.88 ± 2.83	0.005^*^↓	0.008^*^
0.9	5.50 ± 3.03	7.00 ± 2.74	0.002^*^↓	0.007^*^

*The parameters in the table are expressed in the form of mean ± standard deviation. Compared with the TD control group, the down arrow (↓) represents a significant decrease in the ASD group*.

* *p <0.05*.

**Figure 8:**
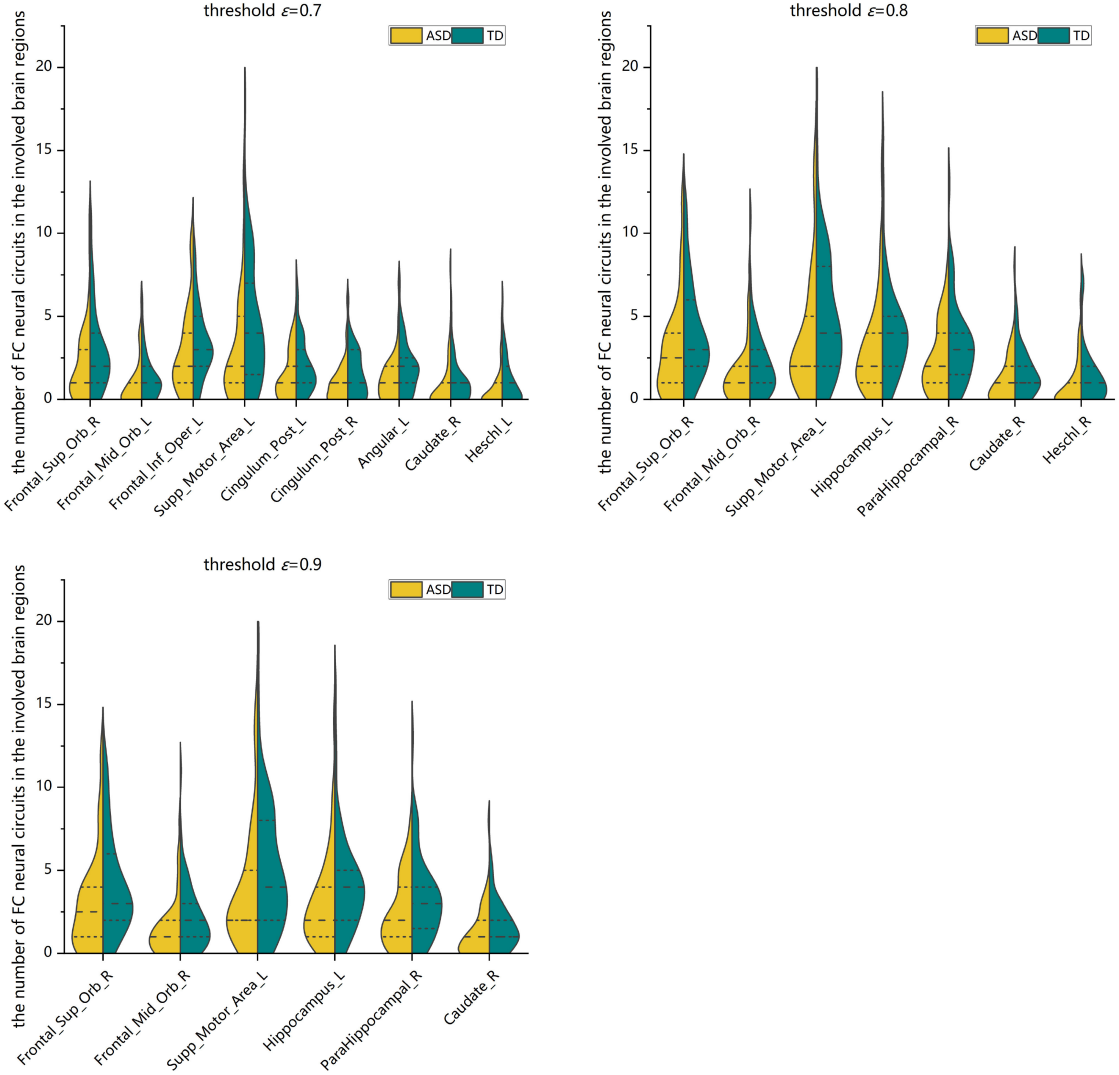
Violin pictures of the comparison of the numbers of FC neural circuits in significantly different brain regions involved in the two groups.

Two-sample Mann-Whitney *U*-tests are performed on the data from the ASD group and the TD control group to assess the group differences in the number of FC neural circuits in each involved brain region. FDR adjustments are performed. The statistical results show that at different threshold values, there are significant differences in the numbers of FC neural circuits in some involved brain regions. Compared with the TD control group, at different threshold values, the number of FC neural circuits in some involved brain regions is significantly decreased in the ASD group. These brain regions include the right orbital part of the superior frontal gyrus, bilateral orbital part of the middle frontal gyrus, left opercular part of the inferior frontal gyrus, left supplementary motor area, bilateral posterior cingulate gyrus, left hippocampus, right parahippocampal gyrus, left angular gyrus, right caudate nucleus, and bilateral Heschl gyrus (see Figure 8 and Table 2).

**Table 2:** Statistically significant differences in the numbers of FC neural circuits in the involved brain regions of the two groups.

**Region**_**ID**	**Threshold value**	**ASD (*n* = 54)**	**TD (*n* = 52)**	***P*-value**	**FDR adjustment**
Frontal_Sup_Orb_R	0.7	2.06 ± 2.28	2.96 ± 2.57	0.033 ^*^↓	0.033 ^*^↓
	0.8	2.91 ± 2.95	4.04 ± 2.94	0.021 ^*^↓	0.032 ^*^↓
	0.9	2.93 ± 2.98	4.10 ± 3.02	0.021 ^*^↓	0.032 ^*^↓
Frontal_Mid_Orb_L	0.7	0.91 ± 1.38	1.31 ± 1.35	0.021 ^*^↓	0.063
	0.8	1.50 ± 2.23	1.73 ± 1.74	0.064	0.064
	0.9	1.50 ± 2.23	1.73 ± 1.74	0.064	0.064
Frontal_Mid_Orb_R	0.7	1.04 ± 1.15	1.44 ± 1.33	0.102	0.102
	0.8	1.44 ± 1.71	2.08 ± 2.04	0.040 ^*^↓	0.060
	0.9	1.44 ± 1.71	2.12 ± 2.07	0.038 ^*^↓	0.060
Frontal_Inf_Oper_L	0.7	2.76 ± 2.53	3.37 ± 2.20	0.049 ^*^↓	0.139
	0.8	3.54 ± 2.82	4.04 ± 2.14	0.139	0.139
	0.9	3.59 ± 2.90	4.10 ± 2.13	0.124	0.139
Supp_Motor_Area_L	0.7	2.87 ± 3.23	4.52 ± 3.78	0.010 ^*^↓	0.015 ^*^↓
	0.8	3.67 ± 3.62	5.19 ± 3.93	0.014 ^*^↓	0.015 ^*^↓
	0.9	3.69 ± 3.61	5.19 ± 3.93	0.015 ^*^↓	0.015 ^*^↓
Cingulum_Post_L	0.7	1.41 ± 1.39	2.00 ± 1.67	0.046 ^*^↓	0.139
	0.8	2.00 ± 1.98	2.40 ± 2.12	0.239	0.239
	0.9	2.02 ± 2.00	2.44 ± 2.09	0.196	0.239
Cingulum_Post_R	0.7	0.96 ± 1.21	1.52 ± 1.42	0.029 ^*^↓	0.087
	0.8	1.37 ± 1.42	1.85 ± 2.00	0.215	0.273
	0.9	1.41 ± 1.43	1.85 ± 2.00	0.273	0.273
Hippocampus_L	0.7	2.46 ± 2.63	3.37 ± 3.26	0.077	0.077
	0.8	3.00 ± 2.75	4.15 ± 3.08	0.011 ^*^↓	0.019 ^*^↓
	0.9	3.04 ± 2.75	4.19 ± 3.09	0.013 ^*^↓	0.019 ^*^↓
ParaHippocampal_R	0.7	1.89 ± 1.99	2.82 ± 2.71	0.055	0.056
	0.8	2.39 ± 2.09	3.32 ± 2.57	0.048 ^*^↓	0.056
	0.9	2.39 ± 2.09	3.34 ± 2.58	0.044 ^*^↓	0.056
Angular_L	0.7	1.26 ± 1.38	1.87 ± 1.55	0.020 ^*^↓	0.061
	0.8	1.70 ± 1.46	2.19 ± 1.98	0.166	0.166
	0.9	1.70 ± 1.46	2.21 ± 1.96	0.140	0.166
Caudate_R	0.7	0.72 ± 1.43	1.10 ± 1.33	0.013 ^*^↓	0.021 ^*^↓
	0.8	1.13 ± 1.51	1.63 ± 1.43	0.020 ^*^↓	0.021 ^*^↓
	0.9	1.15 ± 1.50	1.65 ± 1.43	0.021 ^*^↓	0.021 ^*^↓
Heschl_L	0.7	0.61 ± 1.12	1.04 ± 1.36	0.044 ^*^↓	0.131
	0.8	0.85 ± 1.19	1.27 ± 1.55	0.118	0.137
	0.9	0.87 ± 1.20	1.29 ± 1.63	0.137	0.137
Heschl_R	0.7	0.74 ± 1.22	1.19 ± 1.80	0.144	0.144
	0.8	0.85 ± 1.37	1.35 ± 1.76	0.040 ^*^↓	0.081
	0.9	0.87 ± 1.36	1.35 ± 1.76	0.054	0.081

*The parameters in the table are expressed in the form of mean ± standard deviation. Compared with the TD control group, a down arrow (↓) represents a significant decrease in the ASD group*.

* *p <0.05*.

## 4. Discussion

The lengths and number of FC neural circuits determine the efficiency of information transmission processing. By comparative studies, it was found that the mid-length FC neural circuits of spontaneous activities in the ASD group were significantly decreased compared with those in the TD control group, which is speculated to result in the weakened ability of information interaction between brain regions in ASD patients.

### 4.1. The Frontal Lobes

The frontal lobes participate in high levels of cognition and are extremely important for the development of social behavior. The orbitofrontal cortex, located on the ventral side of the frontal lobe, plays an important role in social emotional cognition and self-regulating behavior in social communication. This provides a basis for effective social communication. Some scholars (Mori et al., 2013; Jonker et al., 2015) believe that neurological functional deficits in the orbitofrontal cortex can cause ASD in patients with social function disorders. The posterior cingulate gyrus is an important part of emotional circuits (Miao et al., 2011), which participate in the processes of emotion and self-evaluation. Some scholars (Lai et al., 2010; Lee et al., 2013; Sophia et al., 2013) have found that the functions of the posterior cingulate gyrus are reduced in ASD patients. Loveland et al. (2008) found that the functional activity of brain regions (including the orbitofrontal cortex and the posterior cingulate gyrus) related to the judgment of facial expressions in emotional tasks was decreased in ASD patients. The opercular part of the inferior frontal gyrus is connected to the amygdala through the insula lobe so that when observing emotional expressions, an unconscious imitation of facial expression can promote the subjective emotional experience as well as the understanding of others' emotional intentions, which is closely related to emotional face processing (Fusar-Poli et al., 2009). Compared with the TD control group, the ASD group had abnormally fewer FC neural circuits in some involved brain regions, including the right orbital part of the superior frontal gyrus, bilateral orbital part of the middle frontal gyrus, left opercular part of the inferior frontal gyrus, and bilateral posterior cingulate gyrus. This indicates that there are FC deficits in these brain regions, which may be the cause of the cognitive dysfunction experienced by ASD patients and their inability to effectively implement social interaction behaviors. The supplementary motor area is related to the voluntary movement control of the trunk muscles. Huang et al. (2015) found that the FC deficit in the premotor cortex in ASD patients may be one of the mechanisms of habituate behaviors. The number of FC neural circuits involved in the left supplementary motor area was abnormally decreased in the ASD group. This finding indicates that the dysfunction of planning and executing movement in ASD patients may result in abnormal cognitive integration.

### 4.2. The Temporal Lobes

The temporal lobes process auditory information and participate in emotion and memory functions. The hippocampus is mainly responsible for the storage, conversion, and orientation of long-term memory, which is related to social episodic memory and self-awareness of memory (Yonelinas et al., 2001; Greenberg et al., 2005). In 1937, Papez found that the “parahippocampal gyrus—hippocampus—papillary body—anterior thalamic nucleus—cingulate gyrus—parahippocampal gyrus” formed the hippocampal circuit, which is related to emotion, learning, memory, and other higher functions. Rudie et al. (2012) showed that the hippocampus and the parahippocampal gyrus in ASD patients are strongly connected, and the habituate behaviors of ASD patients are related to FC of the parahippocampal gyrus. Deficts in the parahippocampal gyrus may cause the dysfunction in emotional and cognitive behaviors. Compared with the TD control group, the ASD group had abnormally fewer FC neural circuits in the left hippocampus and the right parahippocampal gyrus. This indicates that memory dysfunction in ASD patients may be a potential cause of social dysfunction. The Heschl gyrus is located on the superior surface of the temporal lobe. The researcher in Long et al. (2018) found that the Heschl gyrus is responsible for processing the complex spectrum of acoustic information, such as pitch, intensity, and timbre. This finding indicates that speech recognition disorder in ASD patients may result in cognitive dysfunctions.

### 4.3. The Parietal Lobes

The parietal lobes participate in memory processing, which is related to sensory and linguistic functions (Roth and Courtney, 2007; Johnson et al., 2011). The angular gyrus is the visual language center, which facilitates the understanding of the meaning of symbols and words. The left angular gyrus connects the visual, auditory, and somatosensory cortices, and various forms of information generate integrated language activities through the function of the angular gyrus (Joseph, 1993; Hofmann et al., 2008). Compared with that in the TD control group, the number of FC neural circuits in the left angular gyrus was abnormally decreased in the ASD group. This may lead to the cognitive dysfunction in ASD patients and the loss of the connection between what they view and what they hear.

### 4.4. The Subcortical Regions

The caudate nucleus (Sesack and Grace, 2010) is a key brain region in the reward network and plays an important role in behavior monitoring. Many scholars (Portmann et al., 2014; Staal, 2015; Qiu et al., 2016) believe that the caudate nucleus is related to the habituated behavior of ASD patients. Compared with that in the TD control group, the number of FC neural circuits in the right caudate nucleus was abnormally decreased in the ASD group. This indicates that the dysfunction of the caudate nucleus is associated with repetitive and stereotypical behaviors in ASD patients.

In addition, this study shows that most brain regions are unilateral rather than bilateral abnormalities, which indicates that there is brain laterality for FC in ASD patients.

In conclusion, the present study analyzes the FC neural circuits of ASD patients aged 6–13 years in the resting state and finds that there are significant differences in the topological properties of the FC neural circuits of ASD patients compared with those of the TD controls. These abilities of information transmission and the functional integration of brain networks are reduced. The number of FC neural circuits with different length ranges and the number of FC neural circuits in some involved brain regions are different, particularly those associated with social cognition, executive functioning, and memory. These abnormalities indicate that impairments in social cognition and social communication abilities in ASD patients are related to abnormal changes in FC neural circuits. The shortcomings of this study are the small sample size and cross-sectional data. In view of these shortcomings, the next step is to increase the sample size for research and analysis on individual longitudinal data. In addition, networks of different scales influence brain networks differently. Therefore, topographic maps of different scales should be combined for network construction to obtain more abundant local and global information. Therefore, more reliable biological markers for the diagnosis of ASD patients can be found, and more effective neuroimaging evidence for treatment will be provided.

## Data Availability Statement

Publicly available datasets were analyzed in this study. This data can be found at: https://fcon_1000.projects.nitrc.org/indi/abide/.

## Author Contributions

DL and SX were responsible for the study concept and design. WZ contributed to the acquisition of the data. XZ assisted with the analyses and interpretation of the findings. DL drafted the initial manuscript. DL, SX, XZ, and WZ critically reviewed the content and approved the final version of the manuscript for publication. All authors contributed to the article and approved the submitted version.

## Funding

This work was supported by the Natural Science Foundation of Shandong Province of China (ZR2020ZD25) and the Soft Science Research Project of Shandong Province of China (Grant No. 2020RKB01671).

## Conflict of Interest

The authors declare that the research was conducted in the absence of any commercial or financial relationships that could be construed as a potential conflict of interest.

## Publisher's Note

All claims expressed in this article are solely those of the authors and do not necessarily represent those of their affiliated organizations, or those of the publisher, the editors and the reviewers. Any product that may be evaluated in this article, or claim that may be made by its manufacturer, is not guaranteed or endorsed by the publisher.
