# Supplementary material for: Analysis of Brain Functional Connectivity Neural Circuits in Children With Autism Based on Persistent Homology
Source: Front Hum Neurosci. 2021 Sep 13;15:745671. doi: 10.3389/fnhum.2021.745671 (PMC8473898; doi:10.3389/fnhum.2021.745671)
Supplement: Supplementary file 1 [file Data_Sheet_1.PDF]

frontiers

Supplementary Material

1 SUPPLEMENTARY DATA

The preprocessing of data mainly includes the following steps:

(1) Removing the first 10 time points from the time series;

(2) Slice timing eliminates the time phase difference generated by scanning each slice;

(3) Realign: Subjects were excluded according to the maximum head motion value. Subjects whose head motion was more than 3 mm and whose rotation angle was more than 3° were excluded;

(4) Bet: Removing skull strip;

(5) T1 coreg to Fun: Anatomical images are registered to fMRI images;

(6) Segmenting by DARTEL;

(7) Regularizing affinely in the European template;

(8) Nuisance covariate regression: Nuisance regressors (white matter (WM), cerebrospinal fluid (CSF), and Global);

(9) Polynomial trend;

(10) Head motion: Friston 24;

(11) Cleaning low-frequency scanner drift and applying bandpass filtering (0.01-0.1);

(12) Normalizing spatial data by DARTEL;

(13) Smoothing by DARTEL;

(14) Defaulting masks based on the prior template in SPM5;

(15) Selecting anatomical automatic labeling (AAL) to define regions of interest (ROIs);

(16) Calculating the FC matrix based on BOLD time series for each pair of brain regions.

Table S1. The abbreviations for names of brain regions in the anatomical automatic labeling (AAL) atlas

number	Regions	Regions	abbr.
1	Precentral_L	Precental gyrus	PreCG.L
2	Precentral_R	Precental gyrus	PreCG.R
3	Frontal_Sup_L	Superior frontal gyrus, dorsolateral	SFGdor.L
4	Frontal_Sup_R	Superior frontal gyrus, dorsolateral	SFGdor.R
5	Frontal_Sup_Orb_L	Superior frontal gyrus, orbital part	ORBsup.L
6	Frontal_Sup_Orb_R	Superior frontal gyrus, orbital part	ORBsup.R
7	Frontal_Mid_L	Middle frontal gyrus	MFG.L
8	Frontal_Mid_R	Middle frontal gyrus	MFG.R
9	Frontal_Mid_Orb_L	Middle frontal gyrus, orbital part	ORBmid.L
10	Frontal_Mid_Orb_R	Middle frontal gyrus, orbital part	ORBmid.R
11	Frontal_Inf_Oper_L	Inferior frontal gyrus, opercular part	IFGoperc.L
12	Frontal_Inf_Oper_R	Inferior frontal gyrus, opercular part	IFGoperc.R
13	Frontal_Inf_Tri_L	Inferior frontal gyrus, triangular part	IFGtriang.L
14	Frontal_Inf_Tri_R	Inferior frontal gyrus, triangular part	IFGtriang.R
15	Frontal_Inf_Orb_L	Inferior frontal gyrus, orbital part	ORBinf.L
16	Frontal_Inf_Orb_R	Inferior frontal gyrus, orbital part	ORBinf.R
17	Rolandic_Oper_L	Rolandic operculum	ROL.L
18	Rolandic_Oper_R	Rolandic operculum	ROL.R
19	Supp_Motor_Area_L	Supplementary motor area	SMA.L
20	Supp_Motor_Area_R	Supplementary motor area	SMA.R
21	Olfactory_L	Olfactory cortex	OLF.L
22	Olfactory_R	Olfactory cortex	OLF.R
23	Frontal_Sup_Medial_L	Superior frontal gyrus, medial	SFGmed.L
24	Frontal_Sup_Medial_R	Superior frontal gyrus, medial	SFGmed.R
25	Frontal_Mid_Orb_L	Superior frontal gyrus, medial orbital	ORBsupmed.L
26	Frontal_Mid_Orb_R	Superior frontal gyrus, medial orbital	ORBsupmed.R
27	Rectus_L	Gyrus rectus	REC.L
28	Rectus_R	Gyrus rectus	REC.R
29	Insula_L	Insula	INS.L
30	Insula_R	Insula	INS.R
31	Cingulum_Ant_L	Anterior cingulate and paracingulate gyri	ACG.L
32	Cingulum_Ant_R	Anterior cingulate and paracingulate gyri	ACG.R
33	Cingulum_Mid_L	Median cingulate and paracingulate gyri	DCG.L
34	Cingulum_Mid_R	Median cingulate and paracingulate gyri	DCG.R
35	Cingulum_Post_L	Posterior cingulate gyrus	PCG.L
36	Cingulum_Post_R	Posterior cingulate gyrus	PCG.R
37	Hippocampus_L	Hippocampus	HIP.L
38	Hippocampus_R	Hippocampus	HIP.R
39	ParaHippocampal_L	Parahippocampal gyrus	PHG.L

40	ParaHippocampal_R	Parahippocampal gyrus	PHG.R
41	Amygdala_L	Amygdala	AMYG.L
42	Amygdala_R	Amygdala	AMYG.R
43	Calcarine_L	Calcarine fissure and surrounding cortex	CAL.L
44	Calcarine_R	Calcarine fissure and surrounding cortex	CAL.R
45	Cuneus_L	Cuneus	CUN.L
46	Cuneus_R	Cuneus	CUN.R
47	Lingual_L	Lingual gyrus	LING.L
48	Lingual_R	Lingual gyrus	LING.R
49	Occipital_Sup_L	Superior occipital gyrus	SOG.L
50	Occipital_Sup_R	Superior occipital gyrus	SOG.R
51	Occipital_Mid_L	Middle occipital gyrus	MOG.L
52	Occipital_Mid_R	Middle occipital gyrus	MOG.R
53	Occipital_Inf_L	Inferior occipital gyrus	IOG.L
54	Occipital_Inf_R	Inferior occipital gyrus	IOG.R
55	Fusiform_L	Fusiform gyrus	FFG.L
56	Fusiform_R	Fusiform gyrus	FFG.R
57	Postcentral_L	Postcentral gyrus	PoCG.L
58	Postcentral_R	Postcentral gyrus	PoCG.R
59	Parietal_Sup_L	Superior parietal gyrus	SPG.L
60	Parietal_Sup_R	Superior parietal gyrus	SPG.R
61	Parietal_Inf_L	Inferior parietal, but supramarginal and angular gyri	IPL.L
62	Parietal_Inf_R	Inferior parietal, but supramarginal and angular gyri	IPL.R
63	SupraMarginal_L	Supramarginal gyrus	SMG.L
64	SupraMarginal_R	Supramarginal gyrus	SMG.R
65	Angular_L	Angular gyrus	ANG.L
66	Angular_R	Angular gyrus	ANG.R
67	Precuneus_L	Precuneus	PCUN.L
68	Precuneus_R	Precuneus	PCUN.R
69	Paracentral_Lobule_L	Paracentral lobule	PCL.L
70	Paracentral_Lobule_R	Paracentral lobule	PCL.R
71	Caudate_L	Caudate nucleus	CAU.L
72	Caudate_R	Caudate nucleus	CAU.R
73	Putamen_L	Lenticular nucleus, putamen	PUT.L
74	Putamen_R	Lenticular nucleus, putamen	PUT.R
75	Pallidum_L	Lenticular nucleus, pallidum	PAL.L
76	Pallidum_R	Lenticular nucleus, pallidum	PAL.R
77	Thalamus_L	Thalamus	THA.L
78	Thalamus_R	Thalamus	THA.R
79	Heschl_L	Heschl gyrus	HES.L
80	Heschl_R	Heschl gyrus	HES.R
81	Temporal_Sup_L	Superior temporal gyrus	STG.L

82	Temporal_Sup_R	Superior temporal gyrus	STG.R
83	Temporal_Pole_Sup_L	Temporal pole: superior temporal gyrus	TPOsup.L
84	Temporal_Pole_Sup_R	Temporal pole: superior temporal gyrus	TPOsup.R
85	Temporal_Mid_L	Middle temporal gyrus	MTG.L
86	Temporal_Mid_R	Middle temporal gyrus	MTG.R
87	Temporal_Pole_Mid_L	Temporal pole: middle temporal gyrus	TPOmid.L
88	Temporal_Pole_Mid_R	Temporal pole: middle temporal gyrus	TPOmid.R
89	Temporal_Inf_L	Inferior temporal gyrus	ITG.L
90	Temporal_Inf_R	Inferior temporal gyrus	ITG.R
